# Nutrient Deprivation Induces Property Variations in Spider Gluey Silk

**DOI:** 10.1371/journal.pone.0088487

**Published:** 2014-02-11

**Authors:** Sean J. Blamires, Vasav Sahni, Ali Dhinojwala, Todd A. Blackledge, I-Min Tso

**Affiliations:** 1 Department of Life Science, Tunghai University, Taichung, Taiwan; 2 Department of Polymer Science, The University of Akron, Akron, Ohio, United States of America; 3 Department of Biology, Integrated Bioscience Program, The University of Akron, Akron, Ohio, United States of America; Estacion Experimental de Zonas Áridas (CSIC), Spain

## Abstract

Understanding the mechanisms facilitating property variability in biological adhesives may promote biomimetic innovations. Spider gluey silks such as the spiral threads in orb webs and the gumfoot threads in cobwebs, both of which comprise of an axial thread coated by glue, are biological adhesives that have variable physical and chemical properties. Studies show that the physical and chemical properties of orb web gluey threads change when spiders are deprived of food. It is, however, unknown whether gumfoot threads undergo similar property variations when under nutritional stress. Here we tested whether protein deprivation induces similar variations in spiral and gumfoot thread morphology and stickiness. We manipulated protein intake for the orb web spider *Nephila clavipes* and the cobweb spider *Latrodectus hesperus* and measured the diameter, glue droplet volume, number of droplets per mm, axial thread width, thread stickiness and adhesive energy of their gluey silks. We found that the gluey silks of both species were stickier when the spiders were deprived of protein than when the spiders were fed protein. In *N. clavipes* a concomitant increase in glue droplet volume was found. Load-extension curves showed that protein deprivation induced glue property variations independent of the axial thread extensions in both species. We predicted that changes in salt composition of the glues were primarily responsible for the changes in stickiness of the silks, although changes in axial thread properties might also contribute. We, additionally, showed that *N. clavipes*' glue changes color under protein deprivation, probably as a consequence of changes to its biochemical composition.

## Introduction

Biological adhesives are reusable and can adapt to variable conditions [1]–[4]. Hence an understanding of the mechanisms facilitating biological adhesion is pivotal to the development of reusable biomimetic adhesives [3]–[6]. The gluey silks of orb web and cobweb spiders are examples of reusable biological adhesives that vary in property in different environments [3], [7]–[11]. Deprivation of certain nutrients can places stress on the synthesis of biological materials, thus organisms may be forced to vary their nutritional investment in certain materials under nutrient deprivation [12]–[15]. For instance, protein deprivation induces spiders to vary the amino acid constituents of their major ampullate (dragline) silks resulting in variations in strength, extensibility, toughness and stiffness [14], [15]. Whether gluey silks experience similar variations under protein deprivation is currently unknown. Since gluey silks interact with major ampullate silks within webs [15], [16], it might be predicted that variations in gluey silk are concomitant with variations in major ampullate silk.

**Figure 1:**
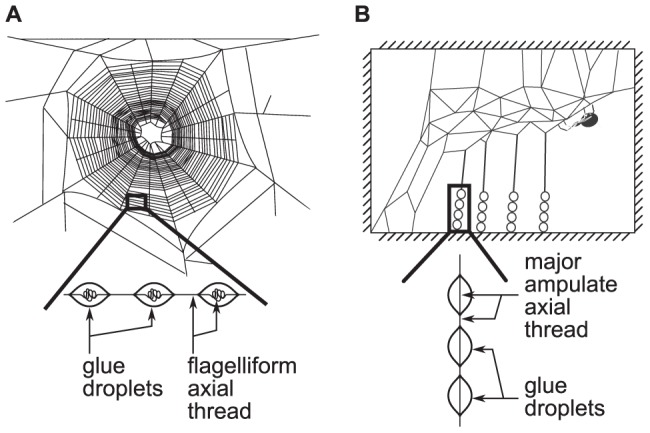
Diagram of the two types of spider gluey silks. Showing: (A) the spiral threads of orb webs and (B) the gumfoot threads of cobwebs. Note that both spiral threads and gumfoot threads consist of a viscoelastic “glue” that forms droplets covering either flagelliform (spiral threads) or major ampullate (gumfoot threads) axial threads.

The gluey silks of most web building spiders function to intercept and retain prey flying or moving at high velocity [17], [18]. There are generally two types of gluey silks produced by web building spiders: (i) the spiral threads added to most orb webs and (ii) the gumfoot threads added to cobwebs [17], [19]. Both spiral threads and gumfoot threads consist of a viscoelastic “glue”, secreted from the aggregate gland of the spider, covering a single (spiral threads) or paired (gumfoot threads) axial threads. In spiral and gumfoot threads the glue coalesces under surface forces into droplets that disperse along the axial thread [5], [20], [21] (Fig. 1). The glues of both types of thread are comprised of an aqueous solution of glycoproteins and low molecular weight organic and inorganic salts [3], [17]. Gumfoot glue, however, contains additional water-soluble peptides [5], [16], [22], [23]. The orb web axial thread is comprised of extensible flagelliform silk [5], [19], [22], while gumfoot axial threads consist of stiffer major ampullate gland (MA) threads [23]. The covering of the axial threads with the aqueous glue causes the flagelliform silk to considerably shrink and increase in extensibility. This enables the kinetic energy of impacting prey to be imparted onto the web, reducing the probability of the prey bouncing off the web [24], [25]. The mechanical properties of spiral and gumfoot threads differ, most likely as a consequence of the different properties of the respective axial silks [19], [26]–[28], although differences in the biochemistries of the glues may also play a role [29].

The spiral threads encompass much of the prey capture area of orb webs [18], [19]. The glycoproteins in the droplet core anchor the spiral glues to the flagelliform threads and extend on contact with prey to promote adhesion via a “suspension bridge” effect [9], [22]. The salts in spiral thread glues cause the glue to lose or gain water from the atmosphere when humidity decreases or increases, influencing the fluidity and stickiness of the glycoprotein core [5], [9]. Furthermore, there is evidence that glue composition, thus the stickiness of spiral threads (experimentally measured as the force required to pull the thread off an inanimate substrate [1], [22], [30]), may vary with the satiation state of the spider and a multitude of other ecological factors [7]–[9].

Both orb webs and cobwebs are attached to substrates using silk secreted by the pyriform glands [31]–[33]. In cobwebs the gumfoot threads extend down from the three dimensional cobweb. The glue is deposited on a portion of the thread near the substrate attachment site and functions by adhering to prey crawling along the ground and releasing the thread from the pyriform silk attachment [19]. Upon release from their pyriform attachment gumfoot threads transmit vibrations towards the cobweb to inform the spider that prey has been captured [32]. Major ampullate silk is stiffer than flagelliform silk, so the gumfoot threads are more efficient at transmitting vibrations to the spider than spiral threads [19]. Furthermore, they experience less strain than spiral threads so they absorb less energy upon contact with an insect and all of the contact force is expended in releasing the thread from the pyriform attachment. The faster the release from the attachment, the more efficient prey capture is [32]. Gumfoot glues undergo insignificant water losses or gains across a humidity gradient [5], suggesting that either the type or function of the salts in gumfoot glues differ from those in spiral glues or the peptides present in gumfoot glues but not spiral glues play a role in hydration [5], [22], [23]. In gumfoot glues there is no evidence for a water sequestering role for salts, nor a thread anchoring or suspension bridge function for the glycoprotein core [5]. No studies have investigated whether or not food deprivation alters gumfoot glue properties, but cobweb architectures and the number of gumfoot threads used increases when cobweb spiders are energetically stressed [34]–[36].

Since gumfoot glues seem less susceptible to ecologically-induced changes in property than orb web glues [5], [17], we predicted that they are less likely than orb web glues to change in property under the influence of changes in nutrient uptake by the spider. Accordingly, we investigated whether protein deprivation induces variation in stickiness of the gluey silks of the orb web spider *Nephila clavipes* and the cobweb spider *Latrodectus hesperus* and we compared the mechanisms inducing any variations.

## Methods

### Ethics statement

Ethics clearance was not required to perform this research. Capture permits were not required under US law as collections were made outside of protected areas. We confirm that the collection locations were not privately owned and we did not collect any endangered or protected species.

### Spider collection and pre-experimental treatments

Fifty penultimate instar female *Nephila clavipes* and fifty final instar female *Latrodectus hesperus* were collected from Gainsville, FL. All spiders were examined for mating and/or egg development and those considered to be virgin were transported to the University of Akron OH in individual 90 mm (diameter) ×45 mm (depth) plastic circular containers with perforated plastic lids. We modified the lids by cutting out a 70 mm×70 mm section and gluing fiberglass mesh (spacing <1 mm) screens to their underside for housing and feeding. To facilitate feeding with a micropipette (20 µl) we cut a 20 mm long slit into the mesh screen using a Stanley knife. The following procedures were performed at constant temperature (∼20°C) and humidity (∼50% R.H.).

To standardize parameters, we fed all spiders 20 µl of a 30% w/v glucose solution (prepared following [37]) daily for five days within their containers, beginning the day the spiders arrived in Akron OH; five days was long enough for the ingested nutrients to be incorporated into silk [13], [15], [37]. Following feeding we placed each spider in individual enclosures made from fiber glass and mesh screen (enclosure sizes: *N. clavipes* = 500×500×200 mm, *L. hesperus* = 370×220×240 mm) for three days to build webs, after which we collected 10 samples of spiral (*N. clavipes*) and gumfoot (*L. hesperus*) threads on 76 mm×26 mm cardboard cards with open-ended 12.7 mm holes punched at one end [38]. Only spiral threads with >12.7 mm spacing between radii (*N. clavipes*: *n* = 46) or gumfoot threads with >6 mm of glue (*L. hesperus*: *n* = 50) were collected. As a result all spirals taken were from the outermost, lower half of the orb webs.

We weighed all spiders before and after the pre-treatment feeding and any spiders (*n* = 2 *N. clavipes*) that lost or gained more than 20% of its mass during pre-feeding were not used in the ensuing experiment.

### Experiment

After the pre-treatment feeding was completed we allocated the spiders to one of two feeding treatments for a further 5 days: (1) a ‘protein fed’ treatment where the spiders were fed daily 20 µl of a mixture of 10 ml of 0.2 g ml^−1^ pure egg albumin solution mixed in a 30 ml of 0.2 g ml^−1^ sucrose solution (see [15] for procedural details) or (2) a ‘protein deprived’, i.e. 20 µl of the 0.2 g ml^−1^ sucrose solution daily (n = 24 per treatment for *N. clavipes* and n = 25 per treatment for *L. hesperus*). Upon feeding we reweighed all of the spiders and placed them in individual cages to build webs over three days before collecting 10 subsamples of spiral (*N. clavipes*) and gumfoot (*L. hesperus*) threads from each web that was feasible to collect from (*N. clavipes*: protein deprived treatment, n = 22, protein fed treatment, n = 21; *L. hesperus*: protein deprived treatment, *n* = 24, protein fed treatment, *n* = 23) on cardboard cards.

### Droplet and axial thread morphological measurements

**Figure 2:**
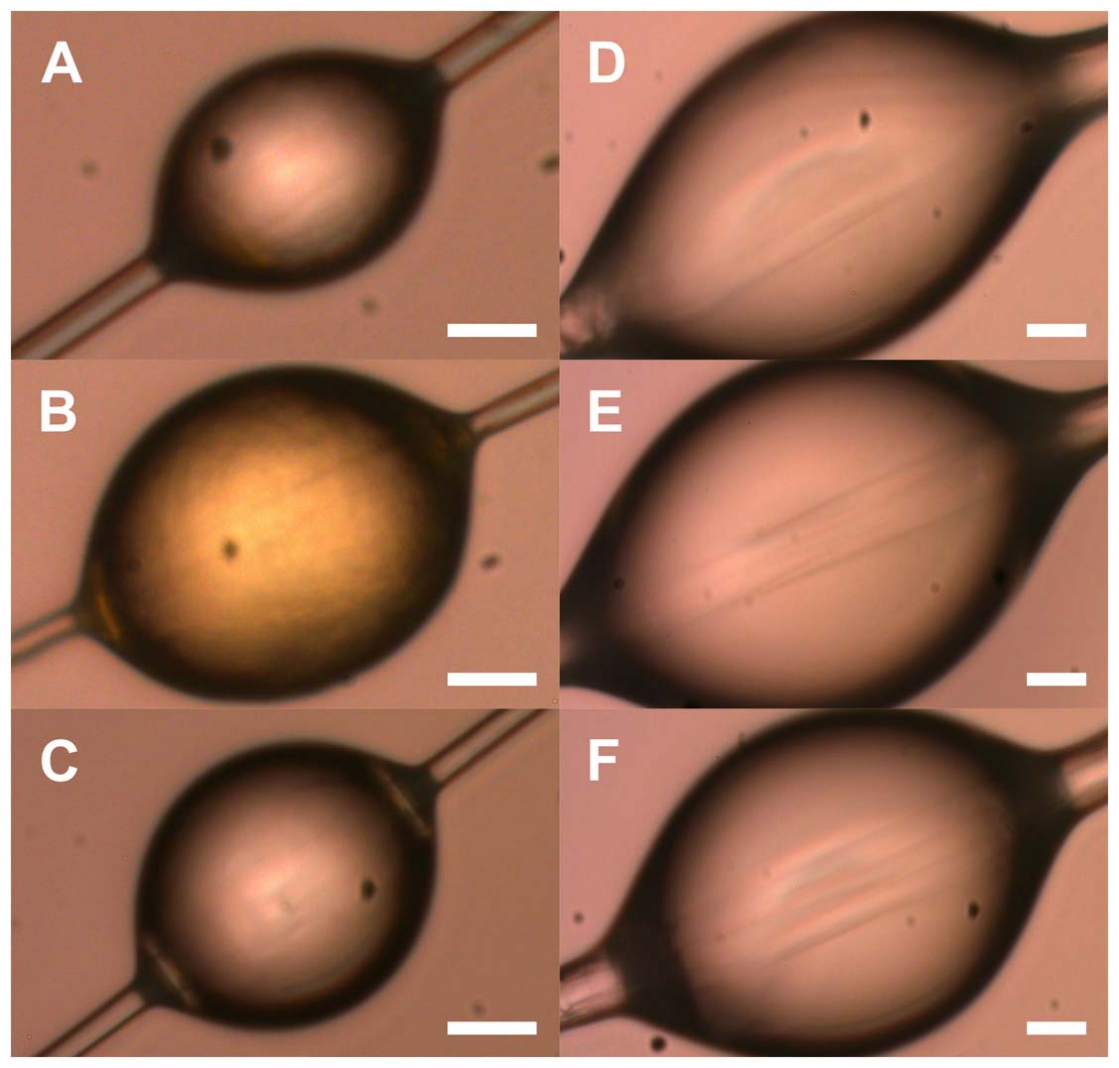
Photographs showing the size of typical glue droplets. Showing: *Nephila clavipes* (A) pre-treatment, (B) when fed a protein deprived solution, and (C) when fed protein, and *Latrodectus hesperus* (D) pre-treatment, (E) when fed a protein deprived solution, and (F) when fed protein. The scale line represents 5 µm. The photographs show *N. clavipes*' droplets increase in volume and change color post-treatment when fed protein, while *L. hesperus*' droplets did not significantly change across treatments.



We subjected five spiral and gumfoot thread subsamples from each pre- and post-treatment web to the following procedures. First, we trimmed the card borders so that the silks were on frames with an approximately 5 mm border. Then we taped the two edges of the frame onto 1.5 mm thick wooden dowels that were stuck (using superglue) 15 mm apart onto a glass slide so that the silk threads did not touch the slide and distort the shape of the droplets. We examined and photographed the threads at 1000× magnification under a polarized light microscope (Olympus BX-50, Tokyo, Japan) following Blackledge et al. [39]. Examples of the resultant photographs are presented in Fig. 2. We then removed the dowels and re-photographed the threads with the droplets flattened so that we could visualize the underlying axial thread. Five photographs were taken at random locations along each thread with a 5.0 Mpixel digital camera (Olympus Q Color 5, Olympus Corp. Melville, NY). For each image we used the programs Image Pro (Media Cybergenics, Bethesda MD) and Image J (NIH, Bethesda MD) to measure three randomly selected glue droplets. We measured the diameter of the flagelliform (*N. clavipes*) or MA silk (*L. hesperus*) axial threads from the images of the flattened droplets. We calculated the volume of the glue droplets (DV), accounting for the diameter of the underlying axial thread, using the formulae [40]:

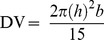
where h is the width of the droplet and b is the length of the droplet.

We, additionally, removed and fixed, using masking tape, each of the frames containing gluey silk threads onto glass slides as described above and examined them under a light microscope at 100× magnification to count the number of droplets running across a randomly selected 1 mm section of thread. We did not photograph the threads at this resolution as no further measurements were made. All morphological measurements were performed within 24 h of collecting the thread. The threads were stored at constant temperature and humidity between collection and measurement to minimize the possibility that the droplet's morphology changed prior to measurement [41].

For, each species, we compared the pre- and post-treatment axial thread diameters, glue droplet volumes and number of glue droplets per mm of thread between protein fed and protein deprived treatments by repeated measures multivariate analyses of variance (rmMANOVAs, with dependent variables: thread diameters, glue droplet volumes and number of glue droplets per mm of thread). Mauchly's tests were used to test for sphericity (p >0.05). Variances were heterogeneous for the pre-treatment and post-treatment *N. clavipes* data (Levene's tests, p<0.05), so these data were square root transformed. Since the rmMANOVAs showed significant overall effects (Table 1), we performed Fisher's Least Significant Difference tests to ascertain the variables that differed across treatments.

**Table 1:** Results of repeated measures (pre-treatment compared to post-treatment) multivariate analyses of variance (rmMANOVA) of droplet morphology (dependent variables: thread diameters, glue droplet volumes and number of glue droplets per mm of thread) between the protein fed and protein deprived treatments for (a) *Nephila clavipes* and (b) *Latrodectus hesperus*.

(a)	df effect	MS effect	df error	MS error	F	p
pre-treatment	1	91.302	40	214.525	0.423	0.655
post-treatment	1	831.682	40	214.603	2.537	0.126
pre-treatment × post-treatment	2	115.364	80	214.603	38.763	<0.001
(b)						
pre-treatment	1	172.272	44	96.079	1.111	0.289
post-treatment	1	114.176	44	102.723	1.793	0.173
pre-treatment × post-treatment	2	684.296	88	102.723	7.124	0.001

### Thread stickiness measurements

We performed the following procedures on a further five spiral and gumfoot thread subsamples from each pre-and post-treatment web sampled within 24 h of collecting the threads. We placed the top (i.e. so the opened end faced downward) of the 76 mm×26 mm cards within the uppermost grips of a UTM Nano Bionix tensile tester (MTS Systems Corporation, Oakridge TN, USA) and a 2 mm glass stage was mounted onto a pin that was held within the lowermost grips. We then lowered the card at 0.01 mm per second until the silk touched the stage. We set the contact force on the stage before holding was initiated to 5 µN for spiral threads and 15 µN for gumfoot threads. Contact was sustained for 60 seconds to allow adhesion of the silk to the glass before the thread was pulled up at 0.1 mm per second until the specimen was pulled off the stage [17]. We plotted load-extension curves for each thread tested using TestWorks 4.0 (MTS Systems Corporation, Eden Prairie MN, USA). The load extension curves were then used to calculate the (1) pull-off force (μN/m^2^); the load at pull-off per unit of glue contact surface, determined by calculating the surface area of the droplets and multiplying it by the number of droplets along 12.7 mm of thread [10], [11], [39], and (2) work at pull-off (*W*_T_) (J/m^2^), according to Sahni et al. [22].

We repeated the above procedures 10 times per sample using a different part of the stage on each occasion, after which an average was calculated. The same procedures were used to measure the stickiness of the spiral threads and gumfoot threads, except we aligned the stage with the middle of the frame for the spiral threads, but for the gumfoot threads we aligned the stage ∼2 mm from the frame border that corresponded to the gumfoot attachment, as the gumfoot glue droplets rarely ran the entire 12.7 mm of thread.

For both species we compared the pre- to post- treatment pull-off forces and *W*_T_ across treatments by repeated measures multivariate analyses of variance (rmMANOVAs, with dependent variables: pull-off force and work at pull-off) upon testing for sphericity (Mauchly's test, p >0.05) and homogeneous variances (Levene's tests, p>0.05). Since the rmMANOVAs showed an overall significant effect (Table 2), we performed Fisher's Least Significant Difference tests to ascertain the variables that differed across treatments. All statistics were carried out using the program SYSTAT 10.2.

**Table 2:** Results of repeated measures (pre-treatment compared to post-treatment) multivariate analyses of variance (rmMANOVA) of thread stickiness (dependent variables: pull-off force and work at pull-off) between the protein fed and protein deprived treatments for (a) *Nephila clavipes* and (b) *Latrodectus hesperus*.

(a)	df effect	MS effect	df error	MS error	F	p
pre-treatment	1	1112.96	40	686.404	1.255	0.268
post-treatment	1	450.267	40	238.981	18.891	<0.001
pre-treatment × post-treatment	2	508.23	80	238.981	37.083	<0.001
(b)						
pre-treatment	1	1118.741	44	997.623	0.708	0.409
post-treatment	1	1284.787	44	185.881	4.206	0.05
pre-treatment × post-treatment	2	901.859	88	185.881	19.94	<0.01

## Results

The body masses of the spiders did not differ pre- compared to post-treatment for either species across treatments (*N. clavipes*: protein fed = 0.381±0.055 g, protein deprived = 0.346±0.042 g, F_1,39_ = 2.84; p = 0.07. *L. hesperus*: protein fed = 0.267±0.081 g, protein deprived = 0.250±0.045 g, F_1,39_ = 0.89; p = 0.41), thus spider mass alone was unlikely to have influenced the following gluey silk property changes.

### Droplet morphology

**Figure 3:**
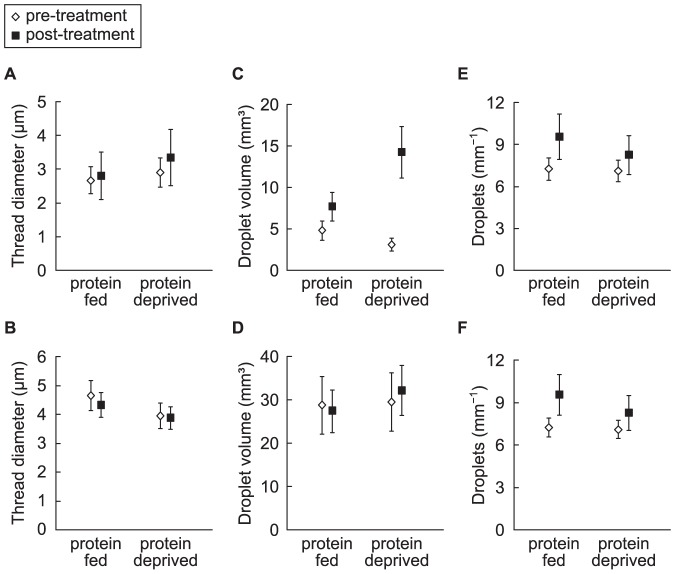
Scatterplot of mean (± s.e) pre-treatment compared to post-treatment droplet morphology values. Showing pre- compared to post-treatment: (A,B) thread diameter, (C,D) glue droplet volume and (E,F) number of droplets per mm of thread values for protein deprived and protein fed (A,C,E) *Nephila clavipes* and (B,D,F) *Latrodectus hesperus*. (*Nephila clavipes*: *n* = 22 protein deprived and *n* = 21 protein fed) (*Latrodectus hesperus*: *n* = 24 protein deprived and *n* = 23 protein fed).

The flagelliform thread diameters (Fisher's Least Significant Difference test, p = 0.44) (Fig. 2) and the number of glue droplets per mm of thread (p = 0.28) did not differ within *N. clavipes* deprived of or fed protein (Fig. 3a,c). Individuals that were deprived of protein, however, had droplets of significantly larger volume than did individuals fed protein (p = 0.02; Fig. 2, 3b). Approximately 64% (67 of 105) of the glue droplets from *N. clavipes* that were deprived of protein appeared yellow-colored, both to the eye and under the polarized microscope (see Fig 2b). We ruled out the possibility that the color change was an anomaly of the microscopy or photography because we did not find similar color changes in any other samples. We did not make biochemical measurements due to the time and logistic constraints of the study, but the composition of some organic compounds, e.g. phenol and quinine, are associated with coloration in spider silks [42], [43], so the observed color changes may have been induced by changes in glue biochemistry.

The major ampullate silk thread diameters (Fisher's Least Significant Difference test, p = 0.42), droplet volume (p = 0.15) (Fig. 2), and the number of droplets per mm of thread (p = 0.11) (Fig. 3c–e), did not differ between protein deprived and protein fed *L. hesperus*.

### Thread stickiness

**Figure 4:**
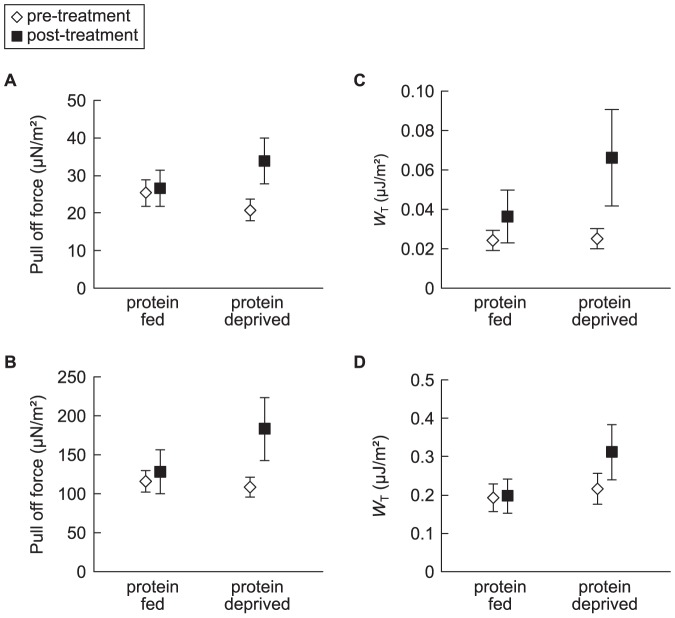
Scatterplot of mean (± s.e) and pull-off force and work at pull-off (*W*_T_). Showing pre- compared to post-treatment: (A,B) pull-off force and (C,D) work at pull-off (*W*_T_) values for protein and protein deprived (A,C) *Nephila clavipes* and (B,D) *Latrodectus hesperus*.

**Figure 5:**
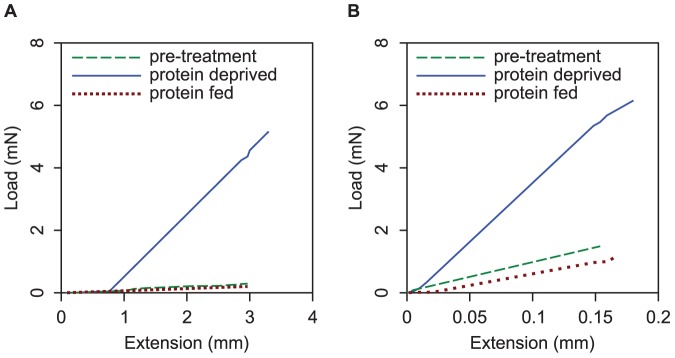
Load extension curves for the gluey silks of pre-treated, protein fed and protein deprived spiders. Showing: (A) representative curves for *Nephila clavipes*, and (B) representative curves for *Latrodectus hesperus*.

We found that, in both species, the thread pull-off force (*N. clavipes*: p = 0.04, *L. hesperus*: p = 0.01; Fig. 4a,b) differed between threads from protein deprived and protein fed spiders. The work done at pull-off, *W*_T_, however differed between protein deprived and protein fed *N. clavipes* (p = 0.02; Fig. 4c) but not protein deprived and protein fed *L. hesperus* (p = 0.36; Fig. 4d). For both species, the slopes of the load-extension curves for the threads from the protein deprived spiders visually differed from those from the protein fed and pre-treated spiders (Fig. 5). This indicates that more force was required to pull the threads of protein deprived spiders off the stage. The extension of the thread at pull-off did not differ between the threads from protein deprived spiders and those from protein fed spiders, suggesting that changes in glue properties but not axial thread properties were responsible for the increased stickiness in the gluey silks of protein deprived spiders.

## Discussion

We found that both *Nephila clavipes* and *Latrodectus hesperus* gluey silks were stickier when the spiders were deprived of protein compared to when the spiders were fed protein. We additionally found that the glue droplets of *N. clavipes*' spiral silks increased in volume when protein deprived but the glue droplets of *L. hesperus*' gumfoot threads did not. This change in glue droplet volume might partly explain why *N. clavipes*' spiral threads were stickier when the spiders were protein deprived. There was a lack of a concomitant increase in glue droplet volume with stickiness in *L. hesperus*' gumfoot threads. Moreover, other studies have found that spiral thread glue droplets may change in volume without affecting stickiness [10], [11], [40]. Hence, we expect the influence of glue volume on the stickiness of either of the gluey threads to be largely inconsequential.

For both gluey silks the thread extension at the time of pull-off did not substantially differ, but the force required to pull the threads off the stage was greater when the spiders were protein deprived. The composition of peptides, glycoproteins, ions and other chelators have been implicated as directly affecting the stickiness of other biological adhesives, such as mussel byssus and insect silks [2], [4], [31]. Accordingly, a change in glue biochemical composition is a plausible explanation why the gluey threads of *N. clavipes* and *L. hesperus* became stickier when protein deprived. Moreover, biochemical variations (e.g. variations in phenol or quinone concentrations) may explain why *N. clavipes*' glue droplets appeared yellow when protein deprived. We did not perform biochemical analyses, but independent analyses of biochemical composition variability in orb web spider glues (Sahni et al. unpubl.) show that glycoprotein composition does not vary substantially within the glues of individuals, but salt compositions may. Thus, it seems plausible that, at least in *N. clavipes*, the increased thread stickiness when protein deprived was facilitated by changes in salt composition. Since salts facilitate water uptake from the atmosphere into spiral glue [5], [26], a change in salt composition may also explain the larger glue droplets found on the spiral threads of protein deprived *N. clavipes*. Changes in salt composition may have, likewise, occurred in the glues of protein deprived *L. hesperus* but without concomitant water uptake or color change. All of the solutions that we fed to spiders were devoid of salt so change in salt composition in the glues of either spider across treatments is unlikely to be a consequence of variations in salt intake.

The work done to detach the thread from the stage, *W*_T_, differed between protein deprived and protein fed *N. clavipes*, but not protein deprived and protein fed *L. hesperus*. The relative contribution of the glue and axial threads to *W*_T_ depends largely on the extension of the axial threads at pull off [22]. In spiral threads the relative contributions of the glue and axial thread to *W*_T_ are estimated to be approximately equal at a thread extension of ∼3 mm [22]; with the glues playing a more substantial role in thread adhesion at extensions less than 3 mm. *L. hesperus* threads never extended to 3 mm and *N. clavipes* spiral threads extended to 3 mm only in extreme cases. Accordingly, the stored adhesive energy in the glue primarily promotes thread stickiness in *L. hesperus*' gumfoot threads. The stored adhesive energy in the extended axial threads, however, would have had more of an influence on thread stickiness in *N. clavipes*' spiral threads, presumably explaining why *W*_T_ differed between protein deprived and protein fed *N. clavipes* but not protein deprived and protein fed *L. hesperus*.

The pull-off force values and the slopes of the load-extension curves showed that more force was required to pull the gluey threads of protein deprived spiders from a stationary position to their detachment from the stage. This suggests that changes in glue properties and not axial thread properties are primarily responsible for the changes in stickiness found in spiral and gumfoot threads when the spiders are under protein deprivation. However, major ampullate silks will become stiffer under protein deprivation [13]. Accordingly, variations in major ampullate silk stiffness may have played an additional role in increasing the stickiness of the gumfoot threads of protein deprived *L. hesperus*. We did not measure the stiffness of the major ampullate silks of the gumfoot threads across treatments so we could not ascertain to what extent changes in thread stiffness influenced the stickiness of gumfoot threads. No studies have been done to assess the influence of protein deprivation on flagelliform silk performance. Nevertheless, the mechanical performance of dry flagelliform silk is similar to major ampullate silk [28] and the extensibility of both silks is strongly dependent on alanine and proline composition [15], [17], [44]–[46], so it is plausible that protein deprivation affects the mechanics of the flagelliform silks, and this may have played a role in inducing an increase in stickiness in the spiral threads of protein deprived *N. clavipes*.

Protein limited spiders need to balance their partitioning of protein among a multitude of physiological and somatic functions, including silk production. Hence spiders that have recently consumed protein might invest more heavily in more nutritiously costly silks [14], [44], [45], [47]. Since the glues contain a comparatively large amount of nutritiously costly compounds, such as the amino acids proline and glutamine, organic and inorganic salts, water-soluble peptides and glycoproteins [16], than major ampullate and flagelliform silk, glue chemical compositions might vary more under nutritional stress. Since the principal function of the glue is the retention of captured prey, while that of the flagelliform or major ampullate axial thread is to absorb prey impacts [18], variability in glue properties may be functionally more tolerable than axial thread property variability. Nevertheless, whether trade-offs between the chemical compositions of gluey silks and other silks exists requires further testing.

An alternative hypothesis posits that the difference in the stickiness of the glues constitutes a change in hunting strategy by the spiders. According to this hypothesis, protein deprived spiders increase their chances of retaining captured prey by increasing their glue stickiness [18]. Given the ability of *N. clavipes*, *L. hesperus* and other spiders to reduce their metabolism and withstand starvation [48], it is unlikely that substantial protein depletion was achieved over the 10 days of our experiment. Hence, the change in hunting strategy hypothesis may be more parsimonious with our results. Our finding of two mechanisms for inducing a change in stickiness in two different spiders suggests possible evolutionary convergence. More information on the likelihood of prey retention in protein fed and protein deprived gluey threads and the physiological means by which spiders might exert control over glue properties is, nonetheless, needed to test this hypothesis.

The spiral glues of *N. clavipes* became yellow when they were protein deprived. Studies show that phenolic and quinone pigments in spiral and dragline silks in *Nephila* spp. are responsible for their gold-colored webs [42], [43], and that *Nephila* web coloration serves to attract insects and may change seasonally [49], [50]. Hence our findings suggest that, in addition to affecting thread adhesiveness, variations in glue biochemistry contributes to the coloration of the webs of *Nephila* spp., and that their webs may change color if the spider is under protein deprivation.

In summary, we found that the size of the glue droplets of *N. clavipes* but not *L. hesperus* increased under protein deprivation and the force and energy required to pull the threads off a surface increased under protein deprivation for both species. The thread extensions at pull off, nevertheless, did not vary across treatment. Since salts promote water uptake from the atmosphere in orb web glues [5], [51] but not gumfoot glues [3], we consider variations in salt composition an explanation of why *N. clavipes* glue droplets' swelled under protein deprivation but *L. hesperus*' glue droplets did not. Since salts can directly or indirectly promote thread stickiness in spiral and gumfoot glues [5], [21], we predict that changes in salt composition explains why spiral and gumfoot threads increased in stickiness when the spiders were protein deprived. Unresolved questions that future studies should answer include: what are the physiological and biochemical mechanisms used to adjust the salt compositions in spider gluey silk? And, is salt investment in gluey silk triggered by nutrient intake or can spiders strategically allocate more or less salts to the glues as their nutritional intake varies?

There is considerable interest in understanding the mechanisms of property variability in gluey silks for biomimetic applications [1]–[6]. We, thus, suggest that future research efforts aim to understand the mechanisms driving sticky silk plasticity at finer scales and under different environmental conditions, e.g. across a wider range of nutrient deprivation.

## References

[1] Gay C (2002) Stickiness-some fundamentals of adhesion. Integr Comp Biol 42: 1123–1126. 21680396 10.1093/icb/42.6.1123

[2] Lee H, Dellatore SM, Miller WM, Messersmith PB (2007) Mussel-inspired surface chemistry for multi-functional coatings. Science 318: 426–430. 17947576 10.1126/science.1147241 PMC2601629

[3] Liu F, Jiang L (2011) Bio-inspired design of multi-scale structures for functional integration. Nano Today 6: 155–175.

[4] Brubaker CE, Messersmith PB (2012) The present and future of biologically-inspired adhesive interfaces and materials. Langmuir 28: 2200–2205. 22224862 10.1021/la300044v

[5] Sahni V, Blackledge TA, Dhinojwala A (2011) Changes in the adhesive properties of spider aggregate glue during the evolution of cobwebs. Sci Rep 1: 41. 22355560 10.1038/srep00041 PMC3216528

[6] Sahni V, Labhasetwar DV, Dhinojwala A (2012) Spider silk inspired microthreads. Langmuir 28: 2206–2210. 22148841 10.1021/la203275x

[7] Townley MA, Tillinghast EK, Neefus CD (2006) Changes in composition of spider orb web sticky droplets with starvation and web removal and synthesis of sticky droplet compounds. J Exp Biol 209: 1463–1486. 16574806 10.1242/jeb.02147 PMC1794320

[8] Opell BD, Lipkey GK, Hendricks ML, Vito ST (2009) Daily and seasonal changes in the stickiness of viscous capture threads in *Argiope aurantia* and *Argiope trifasciata* orb webs. J Exp Zool 311A: 217–225. 10.1002/jez.526 19199347

[9] Opell BD, Karinshak SE, Sigler MA (2011) Humidity affects the extensibility of an orb-weaving spider's viscous thread droplets. J Exp Biol 214: 2988–2993. 21832141 10.1242/jeb.055996

[10] Opell BD, Karinshak SE, Sigler MA (2013) Environmental response and adaptation of glycoprotein glue within the droplets of viscous prey capture threads from araneoid spiders. J Exp Biol 216: 3023–3034. 23619400 10.1242/jeb.084822

[11] Wu CC, Blamires SJ, Wu CL, Tso IM (2013) Wind induces variations in spider web geometry and sticky spiral droplet volume. J Exp Biol 216: 3342–3349. 23737558 10.1242/jeb.083618

[12] Koch AL (1997) Microbial physiology and ecology of slow growth. Micriobiol Mol Biol Rev 61: 305–318. 10.1128/mmbr.61.3.305-318.1997 PMC232613 9293184

[13] Higgins LE, Rankin MA (1999) Nutritional requirements for web synthesis in the tetragnathid spider *Nephila clavipes* . Physiol Entomol 24: 263–270.

[14] Craig CL, Riekel C, Herberstein ME, Weber RS, Kaplan D, Pierce NE (2000) Evidence for diet effects on the composition of silk proteins produced by spiders. Mol Biol Evol 17: 1904–1913. 11110907 10.1093/oxfordjournals.molbev.a026292

[15] Blamires SJ, Wu CL, Tso IM (2012) Variation in protein intake induces variation in spider silk expression. PLoS ONE 7: e31626. 22363691 10.1371/journal.pone.0031626 PMC3282770

[16] Craig CL (2003) Spiderwebs and Silk: Tracing Evolution from Molecules to Genes to Phenotypes. Oxford: Oxford University Press.

[17] Harmer ATM, Blackledge TA, Madin JS, Herberstein ME (2011) High-performance spider webs: integrating biomechanics, ecology and behaviour. J Roy Soc Interf 8: 457–471. 10.1098/rsif.2010.0454 PMC3061126 21036911

[18] Tarakanova A, Buehler MJ (2012) The role of capture spiral silk properties in the diversification of orb webs. J Roy Soc Interf 9: 3240–3248. 10.1098/rsif.2012.0473 PMC3481582 22896566

[19] Peters HW (1987) Fine structure and function of capture threads. In: Nentwig W, editor Ecophysiology of Spiders. Berlin: Springer-Verlag. 187–202.

[20] Opell BD, Hendricks ML (2010) The role of granules within viscous capture threads of orb-weaving spiders. J Exp Biol 213: 339–346. 20038669 10.1242/jeb.036947

[21] Torres FG, Troncoso OP, Cavalie F (2013) Physical characterization of the liquid adhesive from orb-weaving spiders. Mat Sci Eng C 34: 341–344. 10.1016/j.msec.2013.09.030 24268267

[22] Sahni V, Blackledge TA, Dhinojwala A (2010) Viscoelastic solids explain spider web stickiness. Nature Comm 1: 19. 10.1038/ncomms1019 20975677

[23] Hu X, Yuan J, Wang X, Vasanthavada K, Falick AM, et al (2007) Analysis of aqueous glue coating proteins on the silk fibers of the cob weaver, *Latrodectus hesperus* . Biochemistry 46: 3294–3303. 17311422 10.1021/bi602507e

[24] Guinea GV, Cerdeira M, Plaza GR, Elices M, Perez-Riguero J (2010) Recovery in viscid line fibers. Biomacromolecules 11: 1174–1179. 20355706 10.1021/bm901285c

[25] Boutry C, Blackledge TA (2013) Wet webs work better: humidity, supercontraction and the performance of spider orb webs. J Exp Biol 216: 3606–3610. 23788700 10.1242/jeb.084236

[26] Vollrath F, Edmonds DT (1989) Modulation of the mechanical properties of spider silk by coating with water. Nature 340: 305–307.

[27] Swanson BO, Blackledge TA, Hayashi CY (2007) Spider capture silk: performance implications of variation in an exceptional biomaterial. J Exp Zool 307A: 654–666. 10.1002/jez.420 17853401

[28] Blackledge TA, Summers AP, Hayashi CY (2005) Gumfooted lines in black widow cobwebs and the mechanical properties of spider capture silk. Zoology 108: 41–46. 16351953 10.1016/j.zool.2004.11.001

[29] Tillinghast E, Townley MA (1987) Chemistry, physical properties and synthesis of Araneidae orb webs.In: Nentwig W, editor Ecophysiology of Spiders. Berlin: Springer-Verlag. 203–210.

[30] Opell BD (1989) Measuring the stickiness of spider prey capture threads. J Arachnol 17: 112–114.

[31] Perry DJ, Bittencourt D, Liberles-Siltberg J, Rech EL, Lewis RV (2010) Pyriform spider silk sequences reveal unique repetitive elements. Biomacromolecules 11: 3000–3006. 20954740 10.1021/bm1007585 PMC3037428

[32] Sahni V, Harris J, Blackledge TA, Dhinojwala A (2012) Cobweb-weaving spiders produce different attachment discs for locomotion and prey capture. Nat Comm 3: 1106. 10.1038/ncomms2099 23033082

[33] Pugno NM, Cranford SW, Buehler MJ (2013) Synergetic material and structure optimization yields robust spider web anchorages. Small 9: 2747–2756. 23585296 10.1002/smll.201201343

[34] Blackledge TA, Zevenberg JM (2007) Condition-dependent spider web architecture in the western black widow, *Latrodectus hesperus* . Anim Behav 73: 855–864.

[35] Boutry C, Blackledge TA (2008) The common house spider alters the material and mechanical properties of cobweb silk in response to different prey. J Exp Zool 309A: 542–552. 10.1002/jez.487 18651614

[36] Zevenberg JM, Schneider NK, Blackledge TA (2008) Fine dining or fortress? Functional shifts in spider web architecture by the western black widow *Latrodectus hesperus* . Anim Behav 76: 823–829.

[37] Bonthrone KM, Vollrath F, Hunter BK, Sanders JKM (1992) The elasticity of spider's webs is due to water-induced mobility at a molecular level. Proc Roy Soc B 248: 141–144.

[38] Agnarsson I, Blackledge TA (2009) Can a spider web be too sticky? tensile mechanics constrains the evolution of capture spiral stickiness in orb-weaving spiders. J Zool 278: 134–140.

[39] Blackledge TA, Cardullo RA, Hayashi CY (2005) Polarized light microscopy, variability in spider silk diameters, and the mechanical characterization of spider silk. Invert Biol 124: 165–173.

[40] Opell BD, Hendricks ML (2007) Adhesive recruitment by the viscous capture threads of araneoid orb-weaving spiders. J Exp Biol 210: 553–560. 17267640 10.1242/jeb.02682

[41] Opell BD, Schwend HS (2008) Persistent stickiness of viscous capture threads produced by araneoid orb-weaving spiders. J Exp Zool 309A: 11–16. 10.1002/jez.426 18095325

[42] Pouchkina NN, Stanchev BS, McQueen-Mason SJ (2003) From EST sequence to spider silk spinning: identification and molecular characterization of *Nephila senegalensis* major ampullate gland peroxidase NsPox. Insect Biochem Mol Biol 33: 229–338. 12535681 10.1016/s0965-1748(02)00207-2

[43] Putthanarat S, Tapadi P, Zarbook S, Miller LD, Eby RK, et al (2004) The color of dragline silk produced in captivity by the spider *Nephila clavipes* . Polymer 45: 1933–1937.

[44] Blamires SJ, Tso IM (2013) Nutrient-mediated architectural plasticity of a predatory trap. PLoS ONE 8: e54558. 23349928 10.1371/journal.pone.0054558 PMC3551802

[45] Zax DB, Armanios DE, Horak S, Malowniak C, Yang Z (2004) Variation of mechanical properties with amino acid content in the silk of *Nephila clavipes* . Biomacromolecules 5: 732–738. 15132654 10.1021/bm034309x

[46] Liu Y, Shao Z, Vollrath F (2008) Elasticity of spider silks. Biomacromolecules 9: 1782–1786. 18529075 10.1021/bm7014174

[47] Mayntz D, Raubenheimer D, Salomon M, Toft S, Simpson SJ (2005) Nutrient-specific foraging in invertebrate predators. Science 307: 111–113. 15637278 10.1126/science.1105493

[48] Foelix RF (1996) The Biology of Spiders, Second Edition. Oxford: Oxford University Press.

[49] Osaki S (1989) Seasonal changes in color in spiders' silk. Acta Arachnol 38: 21–28.

[50] Craig CL, Bernard GD, Coddington JA (1994) Evolutionary shifts in the spectral properties of spider silks. Evolution 48: 287–296. 28568294 10.1111/j.1558-5646.1994.tb01312.x

[51] Vollrath F, Fairbrother WJ, Williams RJP, Tillinghast EK, Bernstein DT, et al (1990) Compounds in the droplets of the orb spider's viscid spiral. Nature 345: 526–528.

